# The Effect of Yijinjing on the Cognitive Function of Patients With Chronic Schizophrenia

**DOI:** 10.3389/fpsyt.2021.739364

**Published:** 2021-10-20

**Authors:** Hui Gao, Chao Luo, Si-Jing Tu, Ru-Ping Lu, Lin-Na Jiang, Hui-Jun Qiao, Qu Lin, Ning-Ning Li, Jian-Hua Chen

**Affiliations:** ^1^Department of Psychiatry, Shanghai No.1 Mental Health Center of Civil Administration, Shanghai, China; ^2^Shanghai Institute of Traditional Chinese Medicine for Mental Health, Shanghai Clinical Research Center for Mental Health, Shanghai Key Laboratory of Psychotic Disorders, Shanghai Mental Health Center, Shanghai Jiao Tong University School of Medicine, Shanghai, China; ^3^School of Public Health, Hangzhou Normal University, Hangzhou, China; ^4^School of Public Health and Management, Guangxi University of Chinese Medicine, Nanning, China

**Keywords:** aerobic exercise, cognitive function, psychosomatic medicine, Qigong, schizophrenia, traditional Chinese medicine, Yijinjing

## Abstract

**Background:** Patients with chronic schizophrenia present cognitive impairment, which affects their social function and prevents them from reintegrating into society. Yijinjing is a traditional Chinese aerobic exercise that has a putative psychosomatic effect on improving cognitive function.

**Methods:** From January to May 2021, 40 patients with chronic schizophrenia were recruited and randomly divided into a control group and a Yijinjing group. In the 12-week intervention, the patients in the control group received conventional treatment, whereas patients in the Yijinjing group performed Yijinjing exercise (40 min/session, twice a week) in addition to receiving conventional treatment. The Positive and Negative Syndrome Scale (PANSS), the Insight and Treatment Attitude Questionnaire (ITAQ), the Rosenberg Self-esteem Scale (SES), and the Mini Mental State Examination (MMSE) were used to measure clinical symptoms and cognitive function at 0, 6, and 12 weeks.

**Results:** The demographic information was not significantly different between groups. At baseline, the scores of all the scales were not statistically different between groups. After 12 weeks of intervention, compared to those at baseline, the scores of the negative scale (*t* = 19.00, *p* < 0.0001), general psychopathology scale (*t* = 15.98, *p* < 0.0001), and total score (*t* = 15.47, *p* < 0.0001) of the PANSS and SES (*t* = 5.378, *p* < 0.0001) had significantly decreased, and the scores of the ITAQ (*t* = 7.984, *p* < 0.0001) and MMSE (*t* = 6.750, *p* < 0.0001) had significantly increased in Yijinjing group; the score of the MMSE increased in the control group as well (*t* = 2.491, *p* = 0.0222). Compared to the respective scores in the control group, the negative scale score (*t* = 2.953, *p* = 0.0054) significantly decreased, and the ITAQ (*t* = 3.043, *p* = 0.0042) and MMSE (*t* = 2.2.68, *p* = 0.0291) scores significantly increased in the Yijinjing group after 12 weeks of intervention.

**Conclusion:** These results provide a preliminary indication that Yijinjing exercise had the potential to improve cognitive function and negative symptoms in patients with chronic schizophrenia. A larger-scale study to determine the trajectory of change in the longer term should be undertaken.

## Introduction

Schizophrenia is a severe mental disorder that affects 1.13 million people worldwide, and the age-standardized incidence rate in China was 19.81 in 2017 (1, 2). The three core symptoms of schizophrenia are positive symptoms (e.g., hallucinations and delusions), negative symptoms (e.g., blunted affect and avolition), and cognitive impairment (e.g., memory impairment and processing speed inefficiency) (3). Antipsychotics are the first-line medications prescribed for schizophrenia; however, they are accompanied by many adverse effects, such as metabolic dysfunctions, obesity, or even cognitive impairment (4–6). Clinical studies have demonstrated that patients with chronic schizophrenia have a higher risk of metabolic syndromes and worse cognitive function than first-episode patients (7, 8). Self-awareness is a pivotal component of cognitive function, and patients with schizophrenia have severe cognitive dysfunction that is non-specific for this deficiency (9). Therefore, the health management of patients with chronic schizophrenia is a difficult task for psychiatrists and caregivers.

Aerobic exercise can fight metabolic syndrome in obese people and is beneficial to cognitive improvement in people with mental disorders (10, 11). In recent years, accumulating evidence has suggested that physical exercise and physical activity can help to prevent and treat schizophrenia or rehabilitate patients with the disorder (12, 13). Another meta-analysis showed that meditation-based mind–body interventions had potential efficacy in improving mental health, and no serious adverse effects were reported (14). Moreover, Chinese healthy policies encourage sports, especially traditional Chinese exercises, to play an active role in the prevention, treatment, and rehabilitation of chronic diseases (15). By the inspiration of the aforementioned methods and policies, we believed that the integration of exercise and medicine may exert a therapeutic effect on mental disorders.

Yijinjing, similar to Tai Chi or Baduanjin, is a traditional physical exercise in China. The origin of Yijinjing cannot be verified today; the legend states that the Yijinjing was developed based on the movements of the 12 animals by Bodhidharma. Although Yijinjing was invented as a martial art in the first place, it later became a daily exercise method for ordinary people. Literally speaking, the purpose of Yijinjing is to strengthen the flaccid and frail tendons and sinews; in Chinese, *yi* means “change,” *jin* means “tendons and sinews,” whereas *jing* means “methods.” The five core rules of Yijinjing are quietness, slowness, extension, pause, and flexibility. It emphasizes the combination of symmetrical physical postures, meditative mind, and breathing techniques in a harmonious manner (16). Therefore, we hypothesized that Yijinjing integrated with the conventional treatment for schizophrenia may exert a psychosomatic effect in patients with cognitive impairment. To test our hypothesis, a randomized controlled trial was conducted by recruiting chronic schizophrenia patients with stable clinical symptoms and evaluating the improvement of cognitive function after 12 weeks of Yijinjing intervention.

## Materials and Methods

### Study Design and Patients

In accordance with the principles in the Declaration of Helsinki, the study was approved by the ethics committee of Shanghai No. 1 Mental Health Center of Civil Administration (no. YJZXLL2021001) and registered in the Chinese Clinical Trial Registry (no. ChiCTR2100046078). The anonymized patient records were uploaded to the Clinical Trial Management Public Platform. All participants were inpatients from Shanghai No. 1 Mental Health Center of Civil Administration between January and May 2021 and signed the informed consent forms. The hospital is responsible for the rehabilitation of patients with mental disorders, and only for male patients who are unable to return to society and have to maintain therapy. The inclusion criteria were as follows: (a) met the diagnosis criteria of the 10th revision of the *International Classification of Diseases* (*ICD-10*) for schizophrenia, (b) stable mental symptoms, and (c) a disease course of at least 20 years. The exclusion criteria were as follows: (a) comorbidity of other mental disorders, (b) loss of hearing or eyesight, (c) severe cognitive function impairment, and (d) comorbidity of other diseases that did not fit this study, such as physical disabilities or brain lesions.

### Data Collection

Demographic characteristics, including age, sex, course, hospitalization year, years of education, and medication information, were collected at baseline (0 weeks). The antipsychotic doses were converted to equivalent doses of chlorpromazine using the defined daily dose method recommended by the World Health Organization. At 0, 6, and 12 weeks, clinical symptoms were measured by the Positive and Negative Syndrome Scale (PANSS), insight was assessed by the Insight and Treatment Attitude Questionnaire (ITAQ), self-esteem was assessed by the Rosenberg Self-esteem Scale (SES), and global cognitive function was measured by the Mini Mental State Examination (MMSE). The PANSS is used for measuring symptom severity of patients with schizophrenia and recommended by *ICD-10*. The ITAQ, SES, and MMSE were used not only in patients with schizophrenia, but we also used them to interpret the relevant symptoms of the patients. All scales (Chinese versions) have been reported and validated previously (17–19). The scales were evaluated by three senior psychiatrists, and a consistency test was performed with the intraclass correlation coefficient before evaluation.

### Yijinjing Exercise

The patients were randomly assigned to either the control group or the Yijinjing group in accordance with the random number table. The patients in the control group received conventional treatment (e.g., antipsychotic treatment and proper exercise), whereas patients in the Yijinjing group received Yijinjing training in addition to conventional treatment. The Yijinjing exercise was practiced 40 min/d (1 h away from a meal) twice a week for 12 weeks; in the meantime, the patients in the control group were staying in their room, taking a nap, or watching TV. The exercise consisted of four parts: (a) warm-up for 5 min, (b) static exercise for 5 min (meditative mind and breathing practice), (c) Yijinjing exercise for 25 min, and (d) relaxation exercise for 5 min. The exercise was practiced indoors; an exercise coach guided the patients in every part. In the static exercise, the coach led the patients into a meditative state and taking abdominal breathing accompanied by soothing music. The whole set of Yijinjing exercises consisted of 12 postures and was described previously by Xue et al. (20). In the Yijinjing exercise, the patients followed the teaching video, the coach instructed all postures to every participant in the same sequence throughout the trial, and each posture was asked to be as standard as possible based on their ability. After the exercise, the patients were organized for experience exchange and discussion, and the patients were encouraged to practice by themselves every day.

### Statistical Analysis

All of the data are presented as the mean ± SD. IBM SPSS Statistics software was used for data analysis. The Kolmogorov–Smirnov test was used to test for normality. For normally distributed data, intragroup differences were analyzed by paired *t*-tests, whereas intergroup differences were analyzed by *t*-tests. For data that were not normally distributed, group differences were examined using the Kruskal–Wallis test, followed by the Mann–Whitney *U*-test. *p* < 0.05 was considered statistically significant.

## Results

### Background Information of Subjects

Forty patients with chronic schizophrenia who met the inclusion criteria and did not meet the exclusion criteria were invited, and all of them participated in our study with a response rate of 100%. As they were in stable clinical symptoms, they signed the informed consent forms by themselves. Generally, the participants were in middle to old age (range, 37–69 years), with a long course of schizophrenia (range, 20–43 years) and long-term hospitalization (range, 3–38 years). They were randomly divided into a control group (*n* = 20) and a Yijinjing group (*n* = 20), and none of them dropped out during the study. As shown in Table 1, age, sex, course, hospitalization year, years of education, and medication information between groups were not statistically different (all *p* > 0.05).

**Table 1:** Sociodemographic characteristics of patients with chronic schizophrenia.

**Items**	**Control group**	**Yijinjing group**	***t***	***P***
Sex (male)^*^	20 (100)	20 (100)		
Age (years)^#^	53.40 ± 7.04	53.00 ± 8.13	0.166	0.8688
Course (years)^#^	31.75 ± 5.31	31.10 ± 7.63	0.313	0.7562
Hospitalization (years)^#^	12.65 ± 6.48	14.15 ± 8.22	0.641	0.5254
Education (years)^#^	10.95 ± 2.24	9.80 ± 1.82	1.783	0.0826
Antipsychotics				
Aripiprazole^*^	1 (5)	3 (15)		
Chlorpromazine^*^	1 (5)	4 (20)		
Clozapine^*^	6 (30)	2 (10)		
Olanzapine^*^	3 (15)	1 (5)		
Quetiapine^*^	3 (15)	4 (20)		
Risperidone^*^	6 (30)	5 (25)		
Defined daily dose (g)^#^	352.50 ± 117.50	375.00 ± 121.90	0.594	0.5559

* *Reported as n (%)*.

# *Reported as mean ± SD*.

### Scores of Scales in Different Groups

As shown in Table 2, all the scores of the scales were not statistically different between the control group and the Yijinjing group at baseline (all *p* > 0.05). After 6 weeks of intervention, the SES scores decreased (*t* = 4.560, *p* = 0.0005) and MMSE scores increased (*t* = 3.684, *p* = 0.0016) in the Yijinjing group compared to those at baseline. After 12 weeks of intervention, compared to those at baseline, the scores of the negative scale (*t* = 19.00, *p* < 0.0001), general psychopathology scale (*t* = 15.98, *p* < 0.0001), and total scores (*t* = 15.47, *p* < 0.0001) of the PANSS and SES (*t* = 5.378, *p* < 0.0001) had significantly decreased and the scores of the ITAQ (*t* = 7.984, *p* < 0.0001) and MMSE (*t* = 6.750, *p* < 0.0001) had significantly increased in the Yijinjing group; the score of the MMSE increased in the control group as well (*t* = 2.491, *p* = 0.0222). After 12 weeks of intervention, compared to the respective scores in the control group, the negative scale score (*t* = 2.953, *p* = 0.0054) significantly decreased, and the ITAQ (*t* = 3.043, *p* = 0.0042) and MMSE scores (*t* = 2.2.68, *p* = 0.0291) significantly increased in the Yijinjing group.

**Table 2:** Scale scores of two groups in different time point (mean ± SD).

**Scales**	**Control group (*****n*** = **20)**			**Yijinjing group (*****n*** = **20)**		
	**0**	**6**	**12**	**0**	**6**	**12**
PANSS	67.80 ± 15.08	68.50 ± 14.77	66.40 ± 15.55	69.45 ± 13.57	67.70 ± 13.59	58.95 ± 12.39^*^
Positive scale	12.70 ± 6.11	12.40 ± 6.16	12.45 ± 6.59	12.65 ± 4.52	12.25 ± 4.42	12.10 ± 3.95
Negative scale	22.10 ± 4.80	22.80 ± 4.48	21.95 ± 4.76	24.35 ± 5.18	24.00 ± 5.33	17.70 ± 4.33^*^^#^
General psychopathology scale	33.00 ± 7.45	33.30 ± 7.59	32.00 ± 7.28	32.45 ± 6.83	31.45 ± 6.64	29.15 ± 6.56^*^
ITAQ	5.70 ± 5.09	5.65 ± 4.90	5.85 ± 5.17	7.90 ± 6.30	8.45 ± 5.98	11.35 ± 6.21^*^^#^
SES	19.60 ± 1.98	19.40 ± 2.23	19.40 ± 1.93	20.15 ± 1.56	19.15 ± 1.09^*^	18.85 ± 1.18^*^
MMSE	26.80 ± 2.39	27.25 ± 2.29	27.60 ± 2.50^*^	26.85 ± 2.16	27.35 ± 2.32^*^	29.00 ± 1.17^*^^#^

*ITAQ, insight and treatment attitude questionnaire; MMSE, mini mental state examination; PANSS, positive and negative syndrome scale; SES, self-esteem scale*.

* *Vs. 0 weeks, p < 0.05*.

# *Vs. control group, p <0.05*.

## Discussion

In China, primary mental health centers or community medical institutions are the main force for the rehabilitation of patients with chronic schizophrenia (21). Therefore, how and when patients with chronic schizophrenia can regain social function and return to society are primary concerns for doctors and nurses in those institutions. Previous studies have shown that Yijinjing can improve anxiety, depression, and sleep quality for college students (22). A recent clinical trial intended to use Yijinjing to improve the cognitive impairment in post-stroke patients; however, the result is still in progress (20). To the best of our knowledge, we are the first to introduce Yijinjing to schizophrenia patients, and we found that the integration of Yijinjing exercise and conventional treatment for schizophrenia can improve negative symptoms and cognitive function in patients with chronic schizophrenia.

To date, there are many interventions for schizophrenia patients with cognitive impairment, even some third-generation antipsychotics had promising efficacy on cognitive symptomatology associated with schizophrenia (23). The accumulative studies suggested that aerobic exercise seemed most beneficial to cognitive functioning in patients with schizophrenia (24, 25). A meta-analysis showed that aerobic exercise had significant positive effects on preventing age-related hippocampal deterioration, which may be the mechanism for improving cognitive impairment in patients with schizophrenia (26). The exercise type, intensity, and duration of aerobic exercise and the physiology and pathological state of the participant are worth considering (27, 28). Previous studies demonstrated that all intensities of exercise can improve the symptoms and cognitive function of schizophrenia, whereas moderate-intensity showed better benefits (29–31). Yijinjing is a moderate-intensity physical exercise, and the result is in line with previous research. A recent meta-analysis showed that global cognition can be protected by multicomponent exercise in mild cognitive impairment (MCI) patients. Another meta-analysis suggested that aerobic exercise at moderate intensity or above and a total training duration of >24 h can lead to a more pronounced effect on global cognition (32, 33). In our study, the total training duration of Yijinjing was 16 h. The possible explanation for the difference in total training duration was that we only recorded the time when they trained with the coach; perhaps, they practiced on their own in their spare time. Moreover, the integrated interventions targeting both neurocognition and social cognition may optimally improve functional outcomes in schizophrenia (34). The integration of neurocognitive interventions with Yijinjing may be more effective in improving cognitive function; however, it requires further investigation.

Previous studies indicate that traditional Chinese physical exercise (e.g., Tai Chi and Baduanjin) may improve cognitive function in elderly individuals and patients with MCI (35–37). On the other hand, meditation-based mind–body therapies also showed benefits to cognitive function and negative symptoms of schizophrenia (14, 38). Our randomized controlled trial is consistent with the previous studies. The results showed that the Yijinjing exercise can help antipsychotics to improve negative symptoms and schizophrenia symptoms (PANSS), insight (ITAQ), and global cognitive function (MMSE) in patients with chronic schizophrenia. Although the therapeutic mechanism of Yijinjing is still not clear, the advantages of Yijinjing may be as follows: first, unlike aerobic exercise (e.g., jogging or square dancing), Yijinjing exercise emphasizes the meditative and breathing practice, which may relax the mind and mood; second, unlike static exercise (e.g., meditation or Zen), Yijinjing strengthens the body and mind by active working; third, based on the theory of traditional Chinese medicine, Yijinjing can help to promote and circulate the qi and blood through the body. Therefore, Yijinjing exerts the therapeutic effect in a psychosomatic manner.

Given the preliminary nature of this study, the study has limitations that future studies can address. First, the sample size was small, and all the patients were male. Because of the particularity of our hospital, we are a primary mental health center and hospitalized only male patients. We attempted to minimize selection bias using a randomized experimental design, but only 40 patients who met the criteria participated in our study. Fortunately, none of them dropped out during the study. Second, the patients, psychiatrists, and exercise coach could not be blinded, which may have led to performance bias. Third, it is possible that the patients in the Yijinjing group benefited from positive features that are inherent to group-based exercises (e.g., social interactions and attention from exercise coaches). Fourth, as the average age of patients in our study was older than 50 years, and the MMSE is characterized by simplicity and ease of operation, the MMSE was chosen as the cognitive impairment measurement in this study. However, there are some specialized scales for cognitive measurement of patients with schizophrenia, such as the Brief Assessment of Cognition in Schizophrenia and MATRICS Consensus Cognitive Battery, and we may use those measurements in further study (39, 40). Fifth, patients with schizophrenia also have sleep problems such as sleep continuity problems, sleep depth, and REM pressure alterations, which may increase the suicide rate (19, 41). In this study, we paid attention only to the benefits of cognitive functions brought by Yijinjing, while we neglected the adverse effects brought by insomnia, and we would factor in sleep quality in our further trials. In addition, exercise intervention has been reported to relieve antipsychotic-induced metabolic dysfunction (42); however, we did not investigate these aspects.

In conclusion, these results provide a preliminary indication that the integration of Yijinjing exercise and conventional treatment for schizophrenia contributes to the improvement of cognitive function in chronic schizophrenia patients and is sufficiently provocative to warrant further investigation. Thus, a larger-scale study to determine whether Yijinjing would result in improved multidimensional clinical measures of cognitive function should be undertaken in the longer term.

## Data Availability Statement

The original contributions presented in the study are included in the article/supplementary material, further inquiries can be directed to the corresponding author/s.

## Ethics Statement

The studies involving human participants were reviewed and approved by the Ethics Committee of Shanghai No. 1 Mental Health Center of Civil Administration. The patients/participants provided their written informed consent to participate in this study.

## Author Contributions

HG, CL, and J-HC designed the study. HG, R-PL, L-NJ, H-JQ, and QL collected data and performed clinical trial. CL, S-JT, and N-NL analyzed data. HG and CL wrote the main manuscript text. CL and J-HC administrated the study. All authors reviewed the manuscript.

## Funding

This study was supported by the Scientific Research Project of Minhang District Health Committee (2020MW55) and Project of Shanghai Civil Administration.

## Conflict of Interest

The authors declare that the research was conducted in the absence of any commercial or financial relationships that could be construed as a potential conflict of interest.

## Publisher's Note

All claims expressed in this article are solely those of the authors and do not necessarily represent those of their affiliated organizations, or those of the publisher, the editors and the reviewers. Any product that may be evaluated in this article, or claim that may be made by its manufacturer, is not guaranteed or endorsed by the publisher.
